# Burn Wound Care Strategies for the Battlefield and Austere Settings

**DOI:** 10.3390/ebj5010005

**Published:** 2024-02-23

**Authors:** Sarah Shingleton, Jared Folwell, Ian Jones, Michael Gleason, Alicia Williams

**Affiliations:** US Army Institute of Surgical Research, Joint Base San Antonio (JBSA), Fort Sam Houston, TX 78234, USA; jared.s.folwell.mil@health.mil (J.F.); ian.f.jones.mil@health.mil (I.J.); michael.g.gleason3.civ@health.mil (M.G.); alicia.m.williams.mil@health.mil (A.W.)

**Keywords:** burn wound care, burns, austere, prolonged field care, battlefield, combat

## Abstract

Burns are commonly encountered in the battlefield environment; however, the availability of burn expertise and specialized supplies is variable. Initial burn care should remain focused on cooling the burn, preventing hypothermia, basic wound cleansing, and evacuation. Key ongoing burn wound management principles include wound debridement, accurate burn size and depth estimation, wound care, ongoing wound evaluation, and treatment of suspected Gram-negative wound infection. Operative management should be limited to urgent procedures, and definitive burn management should be performed only after evacuation to a higher level of care. Flexibility, creativity, and the ability to adapt care to the tactical environment are key to the successful management of burn injuries in battlefield and austere settings.

## 1. Introduction

Burn injuries are commonly encountered on the battlefield, with an estimated frequency of 5–20% among combat casualties [1,2,3]. The integumentary system is the largest organ in the body and has major homeostatic functions in maintaining body temperature, fluid and electrolyte balance, and protecting against injury and infection. Each of these functions is disrupted in casualties with burns and large traumatic injuries, and the ensuing multisystem response to burn injuries is driven by the wounds. Successful wound management and timely wound closure are essential to patient survival [4]. While timely closure of the burn wound is the primary objective of acute burn care, this may not be feasible in the far forward or prolonged field care (PFC) environment.

The art of burn wound care is a skill developed over years of practice and is one of the most challenging aspects of care. However, expert personnel and specialized resources will likely be unavailable in an austere or far-forward environment. There are many circumstances in which burn care must be provided in an austere environment and under circumstances where usual treatment options are difficult or impossible. Readiness for these circumstances must include creative solutions that are adaptable based on the specific environment.

Key Principles:

- Be prepared to modify how things are performed “back home” and approach burn care with flexibility and common sense [5].
- Family and friends may need to participate in burn care and physical therapy [6].
- Management of burns ≤ 20% total body surface area (TBSA) may need to be managed on an outpatient basis.

## 2. Point of Injury

Burn injuries sustained in combat may be accompanied by blast injuries, penetrating injuries, or other trauma. The initial assessment of any burn casualty should follow the systematic approach laid out in the Advanced Trauma Life Support course (ATLS). This is especially true for combat-related burn injuries, which are predominantly caused by a blast mechanism [7]. Over half of military burn casualties in recent conflicts have presented with additional non-burn injuries [8]. Priority should always be given to immediately life- or limb-threatening injuries.

Initial assessment of burn severity should focus principally on TBSA estimation and less on burn depth [9]. Burn size is a key factor in triage, fluid resuscitation, and determining those who can return to the fight or must be evacuated; therefore, it is important that the provider has a good understanding of how to accurately estimate TBSA burned. Common methods for determining TBSA are the Rule of Nines, the Lund and Browder chart, and the Rule of Hands; mobile applications may also be available. To simplify assessment and reduce error, the estimation tool initially utilized should be the one most familiar to the provider [9,10]. If available, engage remote specialty consultants early [10]. Special attention should be paid to circumferential injuries, particularly full-thickness injuries, as these will require additional monitoring of perfusion to prevent eschar syndrome.

Immediate first aid care of the burn wound should be focused on stopping the burning process, cooling the burn if seen immediately after injury, and providing analgesia [6,9,11]. Potable drinking water may be used to provide burn cooling as well as for rinsing any debris or foreign matter from the wound [12]. In a 2021 systematic review, tap-water cooling for 20 min within 3 h of burn injury resulted in a reduction in burn depth and the need for skin grafting [13]. If readily available, this treatment may be provided by emergency personnel or bystanders. However, extreme care should always be taken to prevent hypothermia, and active rewarming measures should be applied without delay. Ensure that any wound care provided in the field does not delay transport, resuscitation measures, or hypothermia management [1].

Burns from a known or suspected chemical injury should be irrigated copiously with water; any visible dry chemical powders should be brushed away prior to irrigation; and contaminated clothing should be removed [1]. White phosphorous burns are relatively rare but may be encountered during warfare by both military and civilian casualties. Fragments ignite when exposed to oxygen, which often results in visible smoking from the wounds. Wounds should be kept moist with soaking wet dressings or hydrogels. Prompt removal of fragments under continuous irrigation or immersion in cool water may be assisted with the use of a Wood’s lamp to identify fragments [14]. Removed fragments should be placed in a sealable container with cold water for disposal.

Deroofing blisters is controversial and generally not recommended in an emergency setting, but the provider should use clinical judgment [10,15]. Clothing and items such as jewelry should be removed. Appropriate first-aid dressings include a dry, clean cloth or sheet [10]. Plastic cling wrap is also a good alternative; however, ensure that it is not wrapped circumferentially around extremities or the torso [9,16]. Cooling dressings such as moist saline or hydrogel burn dressings should be avoided [6].

Key Principles:

- Life-threatening injuries must be identified and treated prior to managing burn injuries.
- The initial estimation of burn severity should focus on TBSA and not burn depth.
- First-aid wound care consists of cooling the burn while maintaining normothermia and covering the wounds with clean, dry dressings.

## 3. Point of Stabilization

Pain and anxiety are associated with burn wound cleansing, debridement, and dressing changes. Initial pain management is based on frequent, moderate doses of IV narcotics. Ketamine, at a subanesthetic dose, is an excellent agent for painful procedures. Later, an individualized regimen for pain and anxiety management should be instituted. Burn wounds should be cleansed with soap and water at the earliest feasible point and daily thereafter unless placed in an extended-wear dressing.

When possible, debridement should be conducted in the operating theater (OT) to ensure a clean, warm environment [17]. Ideally, an antiseptic such as chlorhexidine should be used [10]. However, under austere conditions, any household soap product may be acceptable [6]. Wounds should be thoroughly cleansed and scrubbed; blisters > 6 mm in diameter should be deroofed; and all loose skin should be debrided. Hair within the burn wound and surrounding area should be shaved if possible. Tweezers and clean washcloths, linen, or gauze may be used to assist with the removal of loose and devitalized tissue [17]. More aggressive debridement performed in the OT may be facilitated by scrub brushes or gauze sponges [17,18]. After this thorough wound debridement, TBSA and burn depth should be reassessed and documented. During wound care, it is important to keep the patient warm, minimize the time wounds are exposed due to large evaporative losses and the risk of hypothermia, and prevent wound desiccation. Strategies to mitigate this risk include exposing only one body part at a time and utilizing thermal blankets to preserve heat.

One complication of burn injuries that does require emergent treatment is eschar syndrome. Any circumferential or near circular full-thickness burn should be identified as early as possible and monitored closely. Indications for extremity escharotomy are changes in the pulse exam, loss of pulse oximetry signals in the affected limb, or the development of neurological deficits [19]. Indications for escharotomies of the chest or abdominal burns can include difficulty with ventilation, hemodynamic instability, and decreased urine output [19]. Providers with the necessary equipment should have a low threshold for performing escharotomies, given the severe consequences of delay. Prophylactic escharotomies should be considered for at-risk patients who are facing long evacuations where escharotomies will not be possible en route.

To perform an escharotomy, longitudinal incisions are made through the burn eschar along the length of the circumferential burn along the medial and lateral sides of a burned extremity or along the midaxillary line on the torso, with an additional incision across the epigastrium as needed. Incisions can be made with a scalpel or electrocautery and should only extend into the subcutaneous fat. The incision should then be inspected by running a finger along it, looking for any dermal bands that will need to be divided. An escharotomy that is properly performed should not cause significant pain or bleeding; any bleeding that does occur can be controlled with electrocautery or topical hemostatic dressings. Perfusion of the extremity should be reevaluated after escharotomy. Patients with an electrical injury or other trauma to the extremity may require additional interventions, such as fasciotomies, to return blood flow to the affected extremity.

Key Principles:

- Use frequent, moderate doses of IV narcotics for initial pain management.
- Wash and debride wounds of devitalized tissue with an antiseptic such as chlorhexidine gluconate.
- Have a strategy to minimize hypothermia.
- Consider prophylactic escharotomies in high-risk patients prior to a long evacuation when it will be difficult to perform en-route.

## 4. Prolonged Wound Management

Burn wounds are dynamic and should be examined frequently to assess infection and wound progression. The timing of reassessment should be based upon patient status and dressing type, i.e., extended-wear dressings require fewer frequent changes. Modifications to the dressing plan of care may be necessary based upon the wound progression, and providers should have a low threshold for early dressing removal in patients who are showing signs of sepsis.

Ideal dressing choices can be challenging, and traditional topical antimicrobials (Table 1) and dressing choices (Table 2) may not be available in enough supply for multiple dressing changes. From a global perspective, the key elements of the ideal burn dressing include the ability to provide non-adherence to the wound bed, reducing the pain of dressing changes, absorbency, and infection prevention [20]. Of these, infection prevention is the most important in combat or disaster casualties. This is accomplished using topical antimicrobials; systemic antibiotics should be reserved for patients with active infections only or another injury that meets indications for prophylaxis (e.g., open fractures). Factors to consider include the level of difficulty involved in caring for the dressing, the condition of the wound and peri-wound, the frequency of dressing changes, and product availability. In cases where the demand for gauze is greater than the available supply, alternative coverings such as clean cloth, feminine napkins, diapers, and pantyhose have been used [6,21]. Alternative dressings that may be available in the far-forward environment that have been described for use in austere settings and developing nations include sterilized banana leaves, aloe vera, and honey, and are discussed in Table 3 [22,23,24,25,26,27].

Silver-impregnated dressings have been successful in the battlefield setting for over 15 years [6]. They are easily applied and can be left in place for up to 7 days per manufacturer guidelines [17]; however, due to infection risk in the battlefield environment, earlier removal and reapplication every 3 days is warranted. Care should be taken to clean and fully debride the burn wounds prior to application because silver dressings are not useful for wounds with heavy contamination. Most dressings must be periodically moistened with potable or sterile water for the silver ion to be active; however, care should be taken not to over moisten which may cause tissue maceration. To prevent hypothermia, a warm environment should be maintained when using moistened dressings. Silver dressings are available in an assortment of sizes, including large sheets and 4- and 6-inch rolls.

Petrolatum-based ointments and antimicrobial creams are typically applied with a non-adherent dressing, followed by gauze wraps or rolls. The open method (creams only) is less ideal but has been shown to be safe and effective [37]. In these instances, reapplication of the topical PRN throughout the day and covering the wounds with a clean, dry sheet is appropriate.

Mafenide acetate 8.5% cream is a topical, short-acting, broad-spectrum antimicrobial. This agent dramatically reduced Gram-negative burn wound infections and associated mortality and has a proven track record in the care of combat casualties [6,38]. Unlike other agents, it penetrates the eschar and other tissues with compromised perfusion. Mafenide acetate is effective against most Gram-negative organisms and multi-resistant organisms; however, it is not effective against yeast, which is one reason it is commonly alternated with 1% silver sulfadiazine cream. If alternating this agent with silver sulfadiazine, it should be applied during the day in a thick layer, followed by a primary gauze dressing and a secondary gauze roll after pre-medicating for pain. When applied to partial-thickness burns, mafenide acetate can be painful.

Silver sulfadiazine cream is a topical agent with antimicrobial properties effective against Gram-positive and Gram-negative bacteria; however, it has poor coverage against *Enterobacter* and some *Pseudomonas aeruginosa*. It is applied in the same manner as mafenide acetate cream at night, as it has a more soothing effect, thereby promoting sleep. Silver sulfadiazine and mafenide acetate creams are labor- and supply-intensive, requiring twice-daily dressing changes; however, they continue to be the primary topical antimicrobial strategy of choice for extensive deep partial- and full-thickness burns (>10% TBSA) at the US Army Burn Center [28].

Cerium nitrate is a rare-earth mineral that has a synergistic effect when combined with silver sulfadiazine, which enhances antipseudomonal activity. It results in a physical hardening and stabilization of the burn eschar, which is particularly useful when early excision is not possible, such as in a PFC environment. In an in vitro porcine skin model, cerium nitrate was shown to reduce colonization and infection caused by *P. aeruginosa* by acting on both the burn eschar and the bacteria directly [39]. It is currently commercially available as a cream in combination with silver sulfadiazine in some countries [6,29] and is applied in the same fashion.

Key Principles:

- Although logistically challenging on the battlefield, alternating 8.5% mafenide acetate and 1% silver sulfadiazine creams is unmatched in efficacy.
- 8.5% mafenide acetate cream should be used for suspected Gram-negative wound infections, if available.
- Traditional wound dressings may not be available in sufficient supply, and improvisation with locally available materials may be necessary.
- Silver-impregnated dressings are less bulky, easier to transport, and reduce the frequency of required dressing changes, but they require prior and complete wound cleansing and debridement.
- Examine wounds frequently and adjust the burn wound management plan as needed.

## 5. Care of Specialized Areas

Most facial burns will heal by assuring a moist wound-healing environment with topical ointments. Facial burns are typically treated via the open method, with twice daily gentle debridement and frequent reapplication throughout the day. Male faces should be shaved daily to avoid folliculitis development. Endotracheal tubes will need to be secured with cotton twill ties because standard tube securement devices will not adhere to burned skin; nasogastric tubes will require similar securement or a nasal bridle [17]. Silicone or foam padding, if available, should be used at the corners of the mouth to prevent pressure injury development [40].

Ear burns require special consideration to prevent severe disfigurement because of the risk of chondritis development. The reduced vascularity of burned ear cartilage is easily damaged, leading to a potential infection. Prevention of trauma, such as pressure from pillows, will help to minimize risk. Twice daily wound cleansing and QID application of 8.5% mafenide acetate cream should be performed [19,41,42].

The hands are of supreme functional importance and are highly vulnerable to the effects of edema, contracture formation, and tendon or joint exposure. Dressings should be constructed so that fingers are wrapped individually, not in the “mitten” style. This will prevent adjacent burned skin from “webbing” together and encourage active range of motion [43]. The feet should be dressed in a similar fashion, with the toes individually wrapped.

Perineal burns may be managed conservatively by twice-daily PRN cleansing and the application of 1% silver sulfadiazine cream. A urinary catheter may be utilized to prevent urine contamination, although this should be weighed against the risk of catheter-associated urinary tract infection [44].

## 6. Skin Substitutes and Off-the-Shelf Products

Several skin substitutes or dermal matrices have been developed in recent years for use in burn centers. These include irradiated human skin allograft (GammaGraft™), decellularized fish skin graft (Kerecis^®^ Omega3), biodegradeable temporizing matrix (BTM; NovoSorb^®^ BTM), polylactic acid skin substitute (Suprathel^®^), and hyaluronic acid ester matrix (Hyalomatrix^®^). These products were all found to be comparable to 1% silver sulfadiazine in terms of wound healing, wound progression, and quantitative bacteriology in deep partial-thickness burns in swine [45]. GammaGraft^TM^ was used in the Combat Support Hospital in Baghdad during the recent conflict in a manner similar to fresh or cryopreserved allograft [46]. But a lack of expertise would likely prohibit the successful use of these products under most circumstances.

## 7. Position and Splinting Techniques

Initial priorities regarding positioning extremities after a burn injury include edema control and pressure relief. Peak swelling usually occurs 12–48 h after a burn [47,48]. Immediate elevation of the extremities, especially burned hands, is essential. The hands should be positioned above the elbows, and the elbows should be positioned at or above the heart. In an austere environment, towel bundles or pillows are readily available items that can be used to elevate the upper extremities [49]. If more aggressive elevation is necessary, surgical netting can be applied to the arm and then attached to an IV pole [49]. Although burn scar contracture prevention is not a priority in the initial days following a burn, anti-contracture positioning is important since the wound will contract over time and could result in loss of function [50].

Splinting is used to prevent/mitigate burn scar contracture, whereby the hand is usually immobilized in a “safe” or intrinsic plus position. The wrist should be positioned in 20° extension, metacarpophalangeal (MCP) joints of the index to small fingers in 70°–90° extension, proximal interphalangeal (PIP) joints and distal interphalangeal (DIP) joints in full extension, and the thumb in palmar abduction. In an austere environment where the typical materials may be limited or unavailable, plaster or aluminum foam material can be used [49]. These materials are useful for the small joints of the hands as well as larger joints such as the ankles, knees, or elbows. Care should be taken to ensure any sharp edges on the aluminum foam are taped or rounded to avoid further tissue injury.

## 8. Operative Burn Management on the Battlefield

The Emergency War Surgery handbook cautions: “Definitive burn surgery with excision and grafting in the combat zone is not advised for patients who can be evacuated to a definitive burn care facility [51].” The provision of surgical care to burn patients in the battlefield environment comes with significant challenges and should normally be limited to necessary procedures such as thorough debridement, escharotomy, and fasciotomy [9]. However, definitive care may be necessary in situations where timely evacuation is impossible, or there is a lack of facilities that can provide definitive burn care to injured civilians. Care decisions should be made in the context of burn size and depth, what is available in the local community, the capabilities of the treatment team, and the tactical situation on the ground.

While assessment of burn size dictates initial burn management, burn depth assessment remains vital in determining appropriate strategies for definitive wound closure. Expert clinical evaluation by a trained burn surgeon remains the accepted standard for assessment of burn depth. However, the accuracy and consistency of clinical exams, even in large burn centers, have been called into question and have sparked further research into technologies to better evaluate burn depth [52]. The challenges of accurate burn depth determination are therefore exacerbated in combat or far-forward situations where expertise is variable and resource limitations abound.

Under normal circumstances, surgical burn wound management hinges on proper depth assessment and the principle of allowing superficial, partial-thickness burns to heal spontaneously, facilitated by good debridement and surgical preparation along with expert local wound care. Excision and grafting should be reserved for deep partial- and full-thickness burns [53].

Burn wounds that have not healed within 3 weeks will necessitate excision and grafting in non-austere conditions [53,54,55]. Early excision and grafting within the first 7 days are the accepted standard of care in large burn centers throughout the developed world, with multiple studies demonstrating improved wound healing, decreased rates of infection, better pain control, and shorter hospitalization [54,55,56,57]. However, this may prove impossible in an austere setting, depending on resources.

It is the opinion of the authors that all efforts to avoid burn excision and grafting in resource-limited environments should be exhausted prior to committing to excision. Moreover, burn wounds should not be excised if an autograft or a skin substitute are not available [17]. However, if it is determined the burns require burn excision and grafting, based on patient, wound, and/or logistical factors, the procedure should be performed in a sterile operating theater, and a staged approach is recommended [17]. While it is ideal to achieve complete burn excision within 1 week, teams not familiar with burn surgery may find it helpful to limit each excision procedure to 10% TBSA [17]. This will help to minimize blood loss and physiologic distress for the patient.

Excision often results in large blood losses that directly correlate with burn surface area, and the team should ensure the availability of adequate blood products to transfuse. Balanced, judicious hemostatic resuscitation is vital to appropriate perioperative management [58]. Furthermore, all technical measures available to mitigate blood loss should be utilized. Additional techniques to minimize surgical blood loss include extremity exsanguination with an Esmarch bandage followed by a pneumatic tourniquet prior to excision, application of topical hemostatic agents such as recombinant thrombin or tranexamic acid following excision or skin harvesting, topical application of a epinephrine solution (1:200,000) to the excised wounds with a non-adherent layer followed by compression, and subcutaneous clysis of donor sites with dilute epinephrine solution (1:1,000.000).

Post-operative wound management should be focused on graft protection and maintaining wound moisture. Many of the aforementioned dressings, with the exception of creams, are appropriate and should generally remain in place for 3–5 days while maintaining limited mobility in areas grafted over joints. Pre-made splints, plaster cast material, or any creative, locally available material may be used to make custom immobility devices. Care should be taken to avoid pressure points.

Enzymatic debridement with bromelain sodium, a proteolytic pineapple-derived enzyme, is a newer, non-surgical debridement technique that is gaining favor for its ability to provide selective debridement in mid- to deep-dermal burns [59]. Benefits include the preservation of viable dermis, a reduction in blood loss, and a reduction in the need for autografting. It has been proposed as a potential field-care burn treatment [60]. However, despite these benefits, there is a steep learning curve, and wound management still requires specialized care by experienced burn teams. Enzymatic debridement is not currently recommended as a “global strategy for mass burn events” [61]; therefore, its role in the battlefield environment still has yet to be determined.

Key Principles:

- For those casualties who can be evacuated out of the combat zone, care on the battlefield remains focused on initial stabilization rather than on definitive care.
- Casualties who cannot be evacuated from the combat zone with ≥50% TBSA burns will likely be triaged into the expectant category.
- Assessment of burn depth is key to formulating a definitive wound management strategy.
- Superficial and indeterminate-depth burns mandate a trial of non-operative management.
- Never excise an uninfected burn without performing coverage with an autograft or a skin substitute at the same operation.
- Conserve blood through restrictive transfusion strategies and utilize measures to minimize blood loss.

## 9. Triage and Wound Care Recommendations Based on Capability

Highlighting again the importance of accurate burn size assessment, casualties with large TBSA deep partial- and full-thickness burns who are unable to be evacuated will have increased mortality due to infection risk, supply availability, number of available beds, and team expertise [6]. Triage of patients to an expectant category may be required if their burns exceed the local capacity to treat and rehabilitate them. The American Burn Association 2014 version 2 triage table [62] is a widely accepted tool and was modified and incorporated into the 2022 European Burns Association burn mass casualty incident (BMCI) guidelines [63]. The recently revised version 3 tables give triage recommendations in a BMCI for conventional, contingency, crisis, and catastrophic burn care, assuming an increasing number of casualties in each level [64]. However, these tables are complex and may not be practical or reliable in all situations.

A small number of burn casualties may overwhelm available resources in an austere setting when evacuation and resupply are not possible. In this situation, a simple method for doing triage is based on the Baux score, defined as the age plus the burn size. For example, a 20-year-old with an 80% TBSA burn has a Baux score of 20 + 80 = 100. At a Baux score of 100, the risk of death in a burn center in a developed nation is currently about 50% [65]. In a BMCI or austere setting in which triage is required, a reasonable approach is to expend scarce resources on patients with a Baux score of 100 or less. This depends on an accurate TBSA calculation to preclude wrongly placing a patient with a survivable burn injury in the expectant category. TBSA is often overestimated by inexperienced personnel. Use the Lund Browder chart carefully. Also take into consideration inhalation injury, medical co-morbidities, and nonburn trauma, all of which can increase mortality. Table 4 summarizes recommended wound care treatments at all management points based on evacuation and resupply capability.

## 10. Conclusions

Burn injuries impact 5–20% of combat casualties, posing unique management challenges. Expert burn care is developed over years of experience and mandates a multi-disciplinary team approach, but access to this level of care is isolated to specialized burn centers. Combat and mass casualty settings frequently develop in remote or austere environs where access to expert burn care is limited or nonexistent. Burn care under these conditions will require flexibility to optimize outcomes. The wound care strategy may require a resourceful approach utilizing the commonsense principles of non-adherent, absorbent, pain-reducing, and infection-mitigating dressings.

While TBSA estimation takes precedence over burn depth during initial evaluation, reassessment of burn depth will guide ongoing wound management. Timely wound closure remains a general priority, but adherence to the principle of spontaneous wound healing of partial-thickness burns, augmented by sound wound care, is critical to patient outcomes and resource conservation under austere conditions. Excision and grafting should be highly restricted to full-thickness or infected burns in casualties who cannot be evacuated in a timely fashion or treated locally, and this should be accomplished in a staged approach to mitigate hemorrhage and the physiologic derangements that accompany burn surgery.

## Figures and Tables

**Table 1:** Topical Antimicrobial and Antifungal Therapies.

Topical Therapy	Activity, Indications and Advantages	Considerations
Petroleum-based ointments	Multiple examples including bacitracin and white petroleum jelly Maintains a moist wound environment Ideal for facial burns and small superficial partial- thickness burns; may be used with healing autografts	Limited to no antimicrobial spectrum of activity May cause local skin irritation with extended use Ophthalmic formulation should be used for periorbital burns
1% silver sulfadiazine cream	Broad coverage against Gram-positive, Gram-negative organisms and yeasts [28] Bactericidal Ideal for deep partial- and full-thickness burns Soothing feeling and easy to use Poor eschar penetration	May cause transient leukopenia Use with caution in patients with sulfa allergy Contributes to pseudo-eschar formation Concentrations >70 ppm result in fibroblast and keratinocyte toxicity resulting in impaired epithelialization
8.5% mafenide acetate cream	Broad coverage against Gram-positive and Gram- negative organisms Bacteriostatic Ideal for full-thickness burns; excellent eschar penetration; Topical of choice for deep ear burns for prevention of chondritis	Potent carbonic anhydrase inhibitor, may cause non-gap metabolic acidosis Painful No fungal coverage and promote fungal growth
Cerium nitrate	Rare earth mineral that interacts with calcium- dependent membrane signaling Bacteriostatic Physical hardening and stabilization of eschar to a “crust”, useful when early excision is not possible	Methemoglobinemia may occur, closely monitor [6] Improvement in burn mortality inconclusive [29] Commercially available in combination with 1% silver sulfadiazine (Flammacerium®, Dermacerium®)
0.5% silver nitrate solution (AgNO3)	Heavy metal; silver ions bind to protein and enzymes Bacteriostatic Good Gram-positive and Gram-negative antimicrobial coverage Dressings must be remoistened frequently [28] Good option for patients with sulfa allergy May be used with negative pressure wound dressing (NPWD) irrigation [30]	Limited wound penetration Is light sensitive, store in appropriate con-tainer Stains upon contact including skin, nails and equipment Hypotonic solution—may result in loss of cations (e.g., hyponatremia and hypochloremia) Rare occurrence of methemoglobinemia
0.125%, 0.25%, 0.5% Sodium hypochlorite solution	Good broad antimicrobial coverage and some fungal coverage Not ideal as a primary dressing due to its immediate onset and **short-lived** action Best used to irrigate contaminated or dirty wounds May be used with NPWD irrigation [30]	Inhibits wound healing—use for short pe-riods of time at the lowest concentration appropriate Is light sensitive, store in appropriate con-tainer
0.5–5% acetic acid solution	Bacteriostatic May be considered for heavily contaminated wounds such as resistant *P. aeruginosa* [31] Daily application as a 10–15 min soak or dressing keeping continuously moist May be used with NPWD irrigation [30]	Inhibits wound healing in vitro studies—use for short periods of time at the lowest concentration appropriate
2% Mupirocin ointment, cream	Inhibits bacterial protein synthesis Effective for Gram-positive organisms Treatment of choice for folliculitis and wounds colonized or infected with *S. aureus*	Rapid resistance, should not be used for more than 10 days
100,000 units/gm Nystatin cream, ointment, powder	Binds to ergosterol and lyses fungal cell membranes Shown to be effective at 6,000,000 units/gm in eradicating fungi in deep wound tissue (however availability is limited) [32]	Powder application followed by moist dressings is easy however tends to cake Cream application maintains a moist wound environment Resistance may occur

**Table 2:** Synthetic Wound Dressings.

Category	Activity, Indications and Advantages	Considerations
Gauze dressings	Coarse or fine mesh gauze dressings Pore size determines absorptive and debridement ability of dressings Primary dressing for creams and wet to moist dressings Mechanical debridement upon removal	Painful upon removal Non-selective debridement and may cause bleeding upon removal
Non-adherent dressings	Multiple examples available Promotes a moist wound environment Ideal over partial-thickness wounds receiving ointments and creams Serves as a contact layer to reduce trauma upon removal Ideal over areas with exposed tendons	Requires a secondary dressing to secure Non-absorptive
Silver impregnated dressings	Available in fabrics that require constant moisture, foams, hydrofibers, alginates Silver-ion-impregnated dressings that provide broad antimicrobial coverage Extended release of silver, varies among products [30]; refer to specific product information Ideal for superficial and deep partial-thickness burns May be left in place for several days	No significant toxicity or resistance Limits the ability to view the wounds daily Absorptive properties of the dressing depend on the base material Follow individual manufacture guidelines for care
Foam dressings	Absorbent dressings Some contain antimicrobials such as silver, polyhexamethylene biguanide and gentian violet/methylene blue Ideal for highly-exudating wounds Reduces wound maceration Comfortable May be left in place for several days Clean wound bed upon removal	Challenging to contour over joints May require secondary dressing to secure May dry wound bed if little exudate
Hydrofiber dressings	Highly absorbent dressings that form a gel layer to maintain a moist wound environment With or without silver Ideal for small, moderate to highly exudative wounds Extended wear times until healed or a change indicated [33]	Limits the ability to visualize the wound bed
Soft-silicone based dressings	Skin-friendly, gentle removal May be a primary contact layer, foam dressing with silicone adhesive or silicone gel sheets Non-adherent to wound bed while gently adhering to the periwound Pain-free removal Leaves no residue on skin Gel sheets ideal for reducing or preventing hypertrophic scarring	Range from minimally absorptive in primary contact layer to highly absorptive if a foam-based dressing Non-absorptive forms will require a secondary dressing
Film dressings	Waterproof but semipermeable to oxygen and water vapor Maintains moist wound environment Prevents wound contamination Transparent allowing for visualization of the wound Ideal for small, superficial wounds	Minimally absorptive and tend to leak with moderate to highly exudative wounds Can tear fragile skin upon removal
Resorbable dressings	Variety available with several containing collagen and/or silver in different formulations Does not require removal from the wound bed May require multiple applications	Absorptive properties vary by dressing type Follow individual manufacture guidelines for care
Negative pressure Wound dressing (NPWD)	Provides a moist environment Promotes granulation tissue formation Instill function compatible with several solutions such as saline, hypochlorite based, biguanides, and acetic acid [30] Provides immobilization of graft onto the wound bed	Assess often to ensure seal if wall suction is used Time consuming and requires high skill level for elaborate dressings Graft, vessels and tendon must be protected with a contact layer Must be removed or replaced if unsealed for greater than 2 h because of infection risk Follow manufacture guidelines for care

**Table 3:** Alternative Austere Wound Dressings.

Alternative	Indications and Advantages	Considerations
Plastic wrap (e.g., cling film)	Protects wound bed from contamination Provides a moist wound environment May be used alone or with ointment	Do not apply circumferentially due to edema and risk for perfusion compromise
Alternatives for gauze bandages	Unscented feminine hygiene pads or diapers Clean cotton cloth Pantyhose [6,21]	Creative securement methods may be needed Cloth dressings may be sterilized and reused
Honey	Ancient wound dressing, natural Available commercially in multiple forms and becoming more mainstream Available in many localities Inflammation suppression, autolytic debridement Gram-positive and Gram-negative coverage Fungal coverage against Fusarium, Aspergilus and Mucor but low MIC for Aspergillus [34]	Pain upon application and challenging to work with Apply with a non-adherent layer Low to moderate level studies showing superiority over 1% silver sulfadiazine cream [24,35] Antibacterial action differs based on type of honey but largely due to high osmolarity and hydrogen peroxide or methylglyoxal
Banana Leaf	Readily available in tropical environments Minimally labor intensive to prepare Can be used with topical ointments Promotes a moist wound healing environment because of wax in the leaves Less painful than gauze during dressing changes [22,27]	Remove the midrib and cut into custom sizes May be adhered to a gauze backing with a flour paste for easier handling Should be sterilized in the autoclave prior to application to ensure no contaminants Slippery, needs to be secured well
Moist exposed burn ointment (MEBO)	Commonly used in Asia and the Middle East for partial-thickness burns Beeswax, sesame oil, and herbal-based with anti- inflammatory and antimicrobial properties Good wound moisture retention	Lacks rigorous studies showing benefits Been shown to be safe with a low complication rate [36]

**Table 4:** Recommendations for Initial, Stabilization, and Prolonged Management Points Based on Capability.

	Immediate Evacuation	Delayed Evacuation	Prolonged Evacuation/Mass Casualty/Austere
Evacuation and Resupply Capability	Evacuation to a burn-capable facility within 24 h of injury	Evacuation to a burn-capable facility within 72 h	Evacuation to a burn-capable facility > 72 h or not available **OR** Austh limited resupply
Triage	Tactical environment situation will be fluid and rapidly changing—triage continuously (see text) Isolated or small number of burn casualties may overwhelm current capabilities in the austere environment [51]		
*Initial Management Point*			
Treatments	First Aid - Stop the burning process - Cool burn with clean water Remove constricting articles and contaminated clothing Trauma management—treat trauma first TBSA determination Hypothermia prevention Pain management Wound management - Leave blisters intact - Cover with clean dry sheet		
*Stabilization Point*			
Treatments	Wash and fully debride—chlorhexidine antiseptic (CHG) preferred Escharotomies if indicated Re-evaluate TBSA Extremity elevation		
Wound considerations	Cover burns with clean dry sheet	≤20% TBSA: any available clean dressings >20% TBSA: extended-wear silver-impregnated dressings Reserve burn creams * for full-thickness burns of intermediate extent (e.g., 40–79% TBSA) [38]	≤20% and ≥80% TBSA: any available clean dressings to include alternative dressings (see Table 3) 21–79% TBSA: extended-wear silver-impregnated dressings (or any available dressings) Reserve burn creams * for burns (any depth) showing clinical signs of infection
*Prolonged Wound Management*			
Treatments	N/A	Reassess wounds with each dressing change Reassess wounds dressed in silver minimally every 3 days Reserve burn creams * for burns (any depth) showing signs of infection Treat suspected wound infection with appropriate IV antibiotics [51] - Erythema beyond a 1 cm margin with other clinical signs of infection - Changes in the burn wound color Extremity elevation Extremity positioning (utilize alternate materials if available)	
Wound considerations	N/A	N/A	If burn excision becomes necessary, perform using a staged technique and limit to 10–20% TBSA per procedure [6,17] Inability to evacuate to definitive care warrants consideration of comfort measures for casualties with a Baux score of >100

* Cerium nitrate-based, mafenide acetate, silver sulfadiazine.

## Data Availability

Not applicable.
